# Comparative Analyses of Phytochemical Variation Within and Between Congeneric Species of Willow Herb, *Epilobium hirsutum* and *E. parviflorum*: Contribution of Environmental Factors

**DOI:** 10.3389/fpls.2020.595190

**Published:** 2021-02-17

**Authors:** Mitra Mohammadi Bazargani, Mohsen Falahati-Anbaran, Jens Rohloff

**Affiliations:** ^1^Agriculture Institute, Iranian Research Organization for Science and Technology, Tehran, Iran; ^2^Department of Plant Sciences, School of Biology, University of Tehran, Tehran, Iran; ^3^NTNU University Museum, Norwegian University of Science and Technology, Trondheim, Norway; ^4^Department of Biology, Norwegian University of Science and Technology, Trondheim, Norway

**Keywords:** bioclimatic variables, *E. hirsutum*, *E. parviflorum*, flavonoid, GC/MS, secondary metabolites, terpenes, steroids

## Abstract

The plants in the *Epilobium* genus are considered to have several important medicinal properties due to their unique chemical composition. Although metabolic profiles of medicinal plants are mainly controlled by genetic factors, their production is also to some degree influenced by environmental factors, thus, variations in the levels of phytochemicals may represent long-term ecological and evolutionary interactions. In order to depict the magnitude of natural variation in level of chemical compounds among conspecific populations of *Epilobium hirsutum* (*n* = 31) and *E. parviflorum* (*n* = 16), metabolite profiling of aerial parts of plants was performed with gas chromatography/mass spectrometry analysis. Putative identification and structure annotation revealed the presence of 74 compounds including 46 compounds considered secondary metabolites categorized into flavonoids (*n* = 8), phenolic acids (*n* = 26), steroids (*n* = 3), and terpenes (*n* = 5) across all populations. Although there was a considerable natural variation among conspecific populations, principal component analysis revealed a clear separation of populations of each species based on the second main principal component which was highly correlated with eight secondary metabolites. The level of secondary metabolites was significantly correlated between species (*r* = 0.91), suggesting shared metabolic pathways underlying the production of chemical compounds. In addition, redundancy and variance partitioning analyses by including bioclimatic variables and altitude revealed a significant contribution of elevation in explaining the total variation of secondary metabolites in *E. hirsutum*. Two-thirds of all secondary metabolites were significantly correlated with altitude in *E. hirsutum*. The large-scale geographic analyses of populations revealed additionally detected flavonoids and terpenes (*E. hirsutum* and *E. parviflorum*) and steroids (*E. hirsutum*) for the first time. This study provides significant information on additional chemical compounds found across the distribution range of the two ecologically important species of willow herb and emphasizes the importance of geographic-wide sampling as a valuable strategy to depict intraspecific and interspecific variability in chemical traits.

## Introduction

An herbal product is economically affordable when the content of secondary metabolites or the so-called specialized metabolites (Tissier et al., 2014) and its active ingredients has reached a desirable and significant level. Both genetic and environmental factors that affect growth and development consequently may alter the biosynthesis of primary and secondary compounds in plants (Moore et al., 2014; Yang et al., 2018). Although these compounds are essentially supervised by genetic processes, their production is also influenced by various environmental factors, and the effect may vary considerably among compounds. The environment is considered an important factor affecting the level of gene expression in pathways involved in biosynthesis of secondary metabolites in medicinal plants. Thus, a large variation in the level of secondary metabolites among populations is expected, as habitat heterogeneity is increased across a larger geographical scale (Falahati-Anbaran et al., 2018).

Although various abiotic environmental factors including geographic features (latitude, altitude), light, and climatic, edaphic, and biotic interactions may affect the content and amount of secondary metabolites in plants, the correlation between the level of secondary metabolites and external variables may reflect the extent of variation and adaptation to specific environmental features (Jaakola and Hohtola, 2010; Gouvea et al., 2012; Sampaio et al., 2016; Demasi et al., 2018; Yang et al., 2018; Li et al., 2020). As shown in the study by Monschein et al. (2015), the phytochemical analysis of aerial tissues of three wild populations of *Epilobium angustifolium* suggested a positive correlation between altitude and the levels of flavanols. To our knowledge, most studies investigating natural variation in the level of secondary metabolites have mostly focused on intraspecific level from limited populations (e.g., Monschein et al., 2015). Although limited studies have also screened the intraspecific variability of secondary metabolites (e.g., Falahati-Anbaran et al., 2018), little is known regarding the interspecific variation in the level of metabolites while comparing multiple populations from a larger geographical range.

The *Epilobium* genus (Onagraceae) consists of nearly 200 species with worldwide distribution, ranging from tropical to temperate regions. Medicinal properties of various species of *Epilobium* have previously been studied. For instance, some *Epilobium* species have been widely used to treat and even prevent a variety of diseases and to enhance wound healing (Sõukand et al., 2020). In addition, the plant has been suggested for the treatment of skin diseases and inflammation such as eczema, acne, burns, and ulcers in traditional medicine (Vogl et al., 2013). The plant has also been successfully applied for bladder health maintenance, male health maintenance, hormonal imbalances, and urinary system health (Constantin et al., 2013).

*Epilobium* plants are rich sources of secondary metabolites, especially polyphenols, such as flavonoids, phenolic acids, and tannins (Granica et al., 2014). Generally, they include two pharmacologically important compounds: flavanols (mainly glycosides of myricetin and quercetin) (Averett et al., 1979; Slacanin et al., 1991; Ducrey et al., 1995; Bazylko et al., 2007a; Hevesi Tóth et al., 2009) and tannins, especially macrocyclic ellagitannins such as oenothein A (OeA) and B (OeB) (Lesuisse et al., 1996; Ducrey et al., 1997; Bazylko et al., 2007b; Shikov et al., 2010). Beside polyphenols, some lipophilic compounds such as steroids (Hiermann and Mayr, 1985; Nowak and Krzaczek, 1998; Pelc et al., 2005; Hevesi Tóth, 2009) and triterpenoids (Glen et al., 1967; Hiermann, 1983; Hevesi Tóth et al., 2006) are also synthesized in the *Epilobium* species.

In recent decades, attention has been increased to understand the chemical composition of *Epilobium*. Although the phytochemical compounds of some species including *E. hirsutum*, *E. angustifolium*, *E. dodonaei*, *E. fleischeri*, *E. roseum*, *E. montanum*, *E. parviflorum*, and *E. tetragonum* has previously been determined (Slacanin et al., 1991; Ducrey et al., 1995; Hiermann, 1995; Hevesi Tóth et al., 2009; Granica et al., 2014), limited knowledge is available on the extent of natural variation among natural populations. In addition, all studies on *Epilobium* have studied chemical composition on a single or few populations within species. Eighteen species of *Epilobium* have been reported in flora of Iran which mainly occur in riparian habitats or moist soil from various geographical regions of Iran (Akbari and Azizian, 2006). Two species, *E*. *hirsutum* and *E. parviflorum*, are native and widely distributed in Iran. These species are growing in a wide range of mountainous regions producing relatively high shoot biomass compared with the other known species in which the size of *E. hirsutum* reaches up to 2 m in some localities (personal observation by authors). The occurrence of *E. hirsutum* and *E. parviflorum* across a wide range of altitudes may suggest adaptation to different ecological conditions and provides an opportunity to perform interspecific comparative analyses of metabolite variation.

Based on our knowledge from extensive literature review, no studies have been done on multiple wild populations of *E. hirsutum* and *E. parviflorum* to demonstrate the extent of natural variation in the content of secondary metabolites and the occurrence of additionally detected chemical compounds. The aims of this study were to quantify the magnitude of natural variation within and among *E. hirsutum* and *E. parviflorum*, two widely distributed species with high shoot biomass. We also tested whether the level of chemical compounds was correlated between species and with environmental variables.

## Materials and Methods

### Plant Material

**FIGURE 1:**
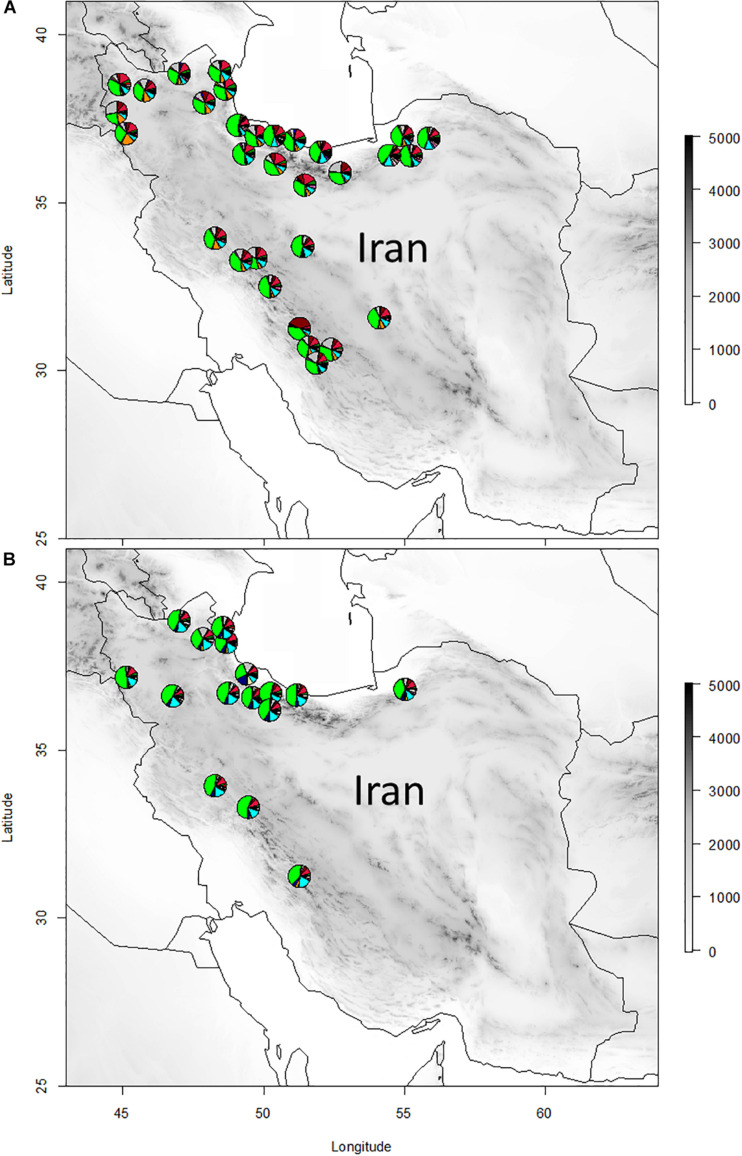
The geographic distribution of natural populations of *Epilobium hirsutum* **(A)** and *E. parviflorum* **(B)** used in the study. Each pie represents the proportion of secondary metabolites for each population. Only those compounds detected at a higher level than 0.25 mg/g DW in at least one population were included (*n* = 31). Gallic acid, quinic acid, 4-hydroxyphenyl ethyl-b-D-glucopyranoside, flavonoid 1 myricetin, and ellagic acid are presented as green, gray, orange, brown, red, and blue color, respectively.

Aerial parts of plants consisting of leaves and flowers from 47 populations of *Epilobium* (Onagraceae) were collected during the flowering period at the end of June from natural habitats, where each individual consisted of at least 50% of opened flowers (Table 1 and Figure 1). The study populations consisted of 31 and 16 natural populations of *E. hirsutum* and *E. parviflorum*, respectively. From each population, the aerial parts of 20 randomly selected individual plants were collected and the samples of each population placed in a paper bag and then air dried at room temperature at ca 30°C in shade. Finally, the plant samples of each population were pooled together and used for phytochemical analysis. It is likely that sample collection and processing on the amount of secondary metabolites and the procedure of air drying may affect compound levels. Since we have used the same drying conditions for all populations, we expect negligible effects of bias caused by air drying on the amount of secondary metabolites.

**TABLE 1:** The geographic information of natural populations of *E. hirsutum* and *E. parviflorum*.

Species	Sample ID	Province	Abbreviation	Locality	Alt. (m a.s.l.)	Latitude (E)	Longitude (N)
*E. hirsutum*	EPH.1	Mazandaran	Nour	Noor-Chamestan	14.6	52.0402	36.52888
	EPH.2	Gilan	Rahi	Rahimabad-Bandbon	88.0	50.3100	37.01185
	EPH.3	Golestan	Zari	Aliabad-Zarin gol	237.4	54.9322	36.89369
	EPH.4	Golestan	Shir	Aliabad-Shirinabad	641.0	55.0277	36.81497
	EPH.6	Golestan	Jira	Jirabad-Shahrood	936.1	55.4601	36.91855
	EPH.7	Golestan	Ziar	Ziaarat	1190.8	54.4649	36.73205
	EPH.8	West Azerbaijan	Ghot	Khoy-Ghotor	1222.4	44.8955	38.52594
	EPH.9	Ardabil	Fand	Fandoghloo	1432.7	48.5359	38.39593
	EPH.10	Gilan	Dam3	Damash 3	1436.0	49.8119	36.77548
	EPH.11	West Azerbaijan	Kali	Kaleybar-Ghale Babak	1470.8	47.0112	38.8508
	EPH.12	Ghazvin	Alam	Alamut-(Dikin)	1544.0	50.4017	36.44972
	EPH.13	Mazandaran	Kela	Kelardasht-Roodbarak	1553.0	51.0897	36.46894
	EPH.14	Gilan	Deyl	Deylaman	1585.4	49.9328	36.89485
	EPH.15	Ardabil	Anba	Anbaran	1710.0	48.4625	38.52436
	EPH.16	West Azerbaijan	Ayne	Ayneroom spring	1719.3	45.1327	37.1828
	EPH.17	Chaharmahal Bakhtiari	Sang	Sandgan spring	1722.0	51.2834	31.25975
	EPH.18	Mazandaran	Java	Javaherdeh	1745.0	50.4756	36.85713
	EPH.19	East Azerbaijan	yam	Marand (Yam)	1747.6	45.7837	38.35466
	EPH.20	Lorestan	Alas	Alashtar (Kahnam)	1760.0	48.2824	33.9435
	EPH.21	West Azerbaijan	Silv	Silvana-Movana	1778.9	44.7861	37.49703
	EPH.22	Ardabil	Kind	Kindarasi-Ardabil	1846.6	47.8990	37.98474
	EPH.23	Kohkiloyeh & Boyerahmad	Yaso	Yasouj	1921.8	51.6045	30.70964
	EPH.24	Lorestan	Alig	Aligudarz	2021.5	49.7406	33.35752
	EPH.25	Tehran	Ahar	Ahar 2	2076.0	51.4692	35.93117
	EPH.26	Fars	Aspa	Fars-Aspas	2141.8	52.3964	30.64337
	EPH.27	Fars	Marg	Margoon waterfall	2146.6	51.8881	30.53075
	EPH.28	Lorestan	Aziz	Azizabad	2210.0	49.5013	33.2846
	EPH.29	Isfahan	Ghaz	Ghazaan-Ghamsar	2224.0	51.3971	33.70588
	EPH.30	Chaharmahal Bakhtiari	Dime	Dimeh	2236.8	50.2273	32.5125
	EPH.32	Tehran	Tang	Tangehvashi	2591.0	52.7266	35.90119
	EPH.34	Yazd	Tara	Dehebala-Tezerjan	2600.0	54.1202	31.58619
*E. parviflorum*	EPP1	Golestan	Alia	Aliabad-Shirinabad	641.0	55.0277	36.81497
	EPP2	Gilan	Chah	Chahar mahal-Barehsar	1118.0	49.7331	36.77707
	EPP3	Ardabil	Hey1	Heyran (Telecabine)	1245.0	48.5801	38.41240
	EPP4	Gilan	Dama	Damash 3	1436.0	49.8119	36.77548
	EPP5	West Azerbaijan	Kali	Kaleybar-Ghale Babak	1470.8	47.0112	38.8508
	EPP6	Ardabil	Hey2	Heyran Tunnel	1510.0	48.5669	38.44718
	EPP8	Mazandaran	Kela	Kelardasht-Roodbarak	1625.3	51.0838	36.46123
	EPP9	West Azerbaijan	Ayne	Ayneroom spring	1719.3	45.1327	37.1828
	EPP10	Chaharmahal Bakhtiari	Sang	Sandgan spring	1722.0	51.2834	31.25975
	EPP11	Lorestan	Alas	Alashtar (Kahnam)	1760.0	48.2824	33.9435
	EPP12	Ghazvin	Ovan	Alamut-Ovan	1857.0	50.4503	36.4980
	EPP13	Lorestan	Aziz	Azizabad	2051.4	49.4577	33.30533
	EPP14	West Azerbaijan	Manb	Manbar-Shahindej	2114.0	46.7681	36.62360
	EPP15	Ghazvin	Alam	Alamut-Station 1	2163.4	50.2175	36.39766
	EPP16	Zanjan	Khan	Khanchai-Taroom	2259.0	48.7480	36.70213
	EPP17	Ardabil	Shab	Shabil	2670.0	47.8438	38.32697

### Extraction of Polar Compounds and Derivatization

Dried plant material was cut into similar small pieces with scissors, and 2 g of tissue was then mixed with 20 ml of 80% methanol (99.8%, anhydrous; Sigma-Aldrich) containing the internal standard ribitol (≥99%; Sigma-Aldrich) at a concentration of 150 μg/ml. Samples were then incubated on a shaking incubator at 25°C for 24 h under fume hood. The extracts were filtered through quantitative and ashless filter paper (Whatman) and centrifuged for 3 min at 3,100 rpm (825 × *g*) at 4°C. The supernatant of each extract was transferred into 1.5 ml round-bottomed microtubes and stored at −20°C until use. Aliquots of sample extracts (500 μl) were dried in a Speed Vac (Savant) overnight (18 h) without heating. Residues were redissolved in 80 μl of 20 mg/ml methoxyamine hydrochloride (98.0%; Sigma-Aldrich) in pyridine (≥99.0%; Sigma-Aldrich) and incubated at 30°C for 90 min. Then, samples were derivatized with 80 μl of *N*-methyl-*N*-(trimethylsilyl) trifluoroacetamide (MSTFA) (≥98.5%; Supelco) by incubation at 37°C for 30 min. Finally, samples were transferred to 1.5 ml autosampler vials with glass inserts and stored at −20°C prior to gas chromatography/mass spectrometry (GC/MS) analysis.

### GC/MS-Based Metabolite Profiling

An Agilent 6890/5975 GC/MS (Agilent Technologies Inc., Palo Alto, CA, United States) was used to analyze all samples. GC separations were carried out using a Supelco SLB 5 ms capillary column (30 m × 0.25 mm and film thickness 0.25 μm). Sample volumes of 1 μl were injected with a split ratio of 15:1. Injection temperature was set at 230°C, and the interface was set to 250°C. The carrier gas used was He at a constant flow rate of 1 ml/min. The GC temperature program was held isothermically at 70°C for 5 min, ramped from 70 to 310°C at a rate of 5°C/min, and finally held at 310°C for 7 min (analysis time: 60 min). The MS source was adjusted to 230°C, and a mass range of *m/z* 70–700 was recorded. All mass spectra were acquired in electron impact ionization (EI) mode (70 eV). Upon visual inspection of GC/MS chromatograms using Agilent ChemStation software (Agilent Technologies, Waldbronn, Germany), raw data was subjected to peak detection, baseline correction, alignment of mass signals, and peak height integration using the data alignment software MetAlign (Wageningen UR, Netherlands). Metabolomics raw data have been deposited to the GNPS database (Global Natural Products Social Molecular Networking, Wang et al., 2016) with the identifier: MassIVE MSV000086585. The complete dataset can be accessed here— doi: 10.25345/C5Z19Z.

### Putative Identification and Structure Annotation of Compounds

Using selected, unique MS intensities, metabolites were semiquantitatively determined as relative metabolite abundance, calculated by normalization of signal intensity to that of ribitol, and concentrations of compounds were finally expressed as milligrams per gram dry weight (DW) (e.g., Lisec et al., 2006). For mass spectral evaluation, deconvolution analysis, and putative identification or structure annotation of metabolite peaks, AMDIS software (National Institute of Standards and Technology, Boulder, CO, United States) was used in combination with the Golm Metabolome database (GMD) (Max-Planck Institute for Molecular Plant Physiology, Golm, Germany), the MassBank high-resolution mass spectral database (NORMAN Association, Verneuil-en-Halatte, France), the NIST05 spectral library (National Institute of Standards and Technology, Gaithersburg, MD, United States), and an *in-house* MS and retention index (RI) library of derivatized plant metabolites [GMD linear retention indices (LRI) and LRI values from literature]. An AMDIS minimum net match factor of ≥80% could be reported for all phytochemicals, which are included with mass spectra in the applied MS libraries (phenolic acids, flavonoids, terpenes, and steroids). The putative characterization and structure annotation of metabolite peaks (aromatic compounds) was based on the presence of known qualifier ions (Rohloff, 2015). In addition, LRI of detected metabolites were recalculated using a homologous series of *n*-alkanes in order to validate putative identifications, based on a combination of mass spectral data and RI values.

### Statistical Analyses

To determine the differences between the study species, the level of major secondary metabolites were compared between *E. hirsutum* and *E. parviflorum* (Supplementary Table 1). In addition, to determine the overall similarity between *E. hirsutum* and *E. parviflorum*, the correlation between the average amount of secondary metabolites (*n* = 46) between species was tested. To illustrate the amount of natural variation within and between species, principal component analysis was performed using XLSTAT 2007.6, and the results were presented as a two-dimensional plot based on two first main components. In addition, the correlation between chemical compounds and the two main principal components were illustrated.

In order to produce a topographic map, elevation data with spatial resolution of 30 s was obtained from WorldClim 2.0 database (Fick and Hijmans, 2017) and plotted on geographic map with raster package in R (Hijmans and Etten, 2020). The chemical composition of populations were presented as a pie chart on the topographic map with Plotrix (Lemon, 2006). The geographic coordinates of each population was used to extract 19 bioclimatic variables from WorldClim 2.0 based on spatial resolution 30 s (Supplementary Table 2). To determine the contribution of bioclimatic variables and altitude in total variation of chemical variables, variance partitioning and redundancy analysis (RDA) were performed on scaled data using *varpart* and *rda* functions with *vegan* in R package, respectively (Oksanen et al., 2019). We applied maximum absolute scaling on both climatic and secondary metabolite data by dividing the value of each observation to the maximum value for a given variable using *decostand* function with *vegan*. RDA is a multivariate analysis that combines multiple linear regressions with classical ordination technique, PCA. The significance of canonical ordinations was tested by means of 1,000 permutations. In order to investigate the relationship between scaled value of phytochemical compounds and altitude, the correlation analysis was performed using XLSTAT 2007.6. This analysis was also performed on scaled data for the major groups of chemical compounds, i.e., flavonoids, phenolic acids, steroids, and triterpenes, separately, and the relationship was further reported as scatter plot.

## Results

### The Number of Chemical Compounds

The results of metabolite profiling with GC/MS analysis in *E. hirsutum* and *E. parviflorum* populations revealed 74 chemical compounds including 46 secondary metabolites (Table 2). Other compounds were mainly classified as primary metabolites (*n* = 18) and non-identifiable aromatic compounds (*n* = 10) putatively characterized based on aromatic qualifier ions, and therefore were less discussed in this paper. In terms of phytochemical properties, 46 compounds were classified into different groups including flavonoids (*n* = 8), phenolic acids and its derivatives (*n* = 26), steroids (*n* = 3), terpenes (four triterpenes and one diterpene), lignin and neolignan glycosides (*n* = 3), and a compound belonging to the group of plant hormones (Table 2).

**TABLE 2:** The average amount of secondary metabolites across populations of *Epilobium hirsutum* and *E. parviflorum* and the relationship between chemical compounds and altitude.

Compound	RT	LRI	LRI cal	*E. hirsutum*			*E. parviflorum*		
				Mean ± SD	CV (%)	*r*	Mean ± SD	CV (%)	*r*
**Flavonoids**									
Epicatechin	48.10	2841.9	2856.5	0.01 ± 0.01	100	–0.19	0.04 ± 0.07	175	0.01
Catechin	49.20	2864.2	2939.3	0.01 ± 0.01	100	0.24	0.01 ± 0.01	100	–0.22
Kaempferol	50.65	3071.9	3052.3	0.54 ± 0.50	93	0.45*	0.76 ± 0.70	92	0.03
Quercetin	51.98	3169.1	3159.7	1.49 ± 1.32	89	0.40*	1.55 ± 1.36	88	–0.04
Myricetin	52.56	3209.6	3207.7	5.01 ± 4.44	89	0.45*	4.72 ± 4.16	88	–0.06
Flavonoid1	52.71		3220.2	3.30 ± 4.46	135	0.41*	1.74 ± 1.80	103	–0.08
Flavonoid2	53.27		3267.4	0.07 ± 0.06	86	0.52**	0.07 ± 0.05	71	–0.01
Kaempferol deriv.	58.79		3771.7	0.01 ± 0.03	300	0.36*	0.01 ± 0.02	200	–0.24
**Phenolic acids and their derivatives**									
2-Phenyl ethanol	15.95	1214.0	1238.2	0.01 ± 0.01	100	0.42*	0.01 ± 0.01	100	–0.03
Benzoic acid	16.56	1251.2	1258.0	0.07 ± 0.06	86	0.45*	0.04 ± 0.03	75	–0.09
Phenylacetic acid	17.99	1303,0	1305,7	0.01 ± 0.01	92	0.49**	0.01 ± 0.01	71	–0.01
4-Hydroxybenzaldehyde	23.22	1493.6	1495.9	0.05 ± 0.04	80	0.53**	0.03 ± 0.03	100	0.01
*p*-Tyrosol (phenolic antioxidant)	25.07	1575.3	1569.6	0.37 ± 0.45	122	0.26	0.08 ± 0.08	100	–0.20
4-Hydroxybenzoic acid	26.40	1633.3	1624.8	0.09 ± 0.07	78	0.41*	0.07 ± 0.07	100	–0.11
Hydroxyquinol	25.73	1598.1	1596.7	0.03 ± 0.03	100	0.61**	0.05 ± 0.04	80	–0.05
4-Hydroxyphenylacetic acid	26.64	1644.0	1635.0	0.01 ± 0.01	100	0.23	0.01 ± 0.01	100	–0.04
2,5-Dihydroxybenzaldehyde	28.20	1709.8	1702.7	0.01 ± 0.01	100	0.40*	0.04 ± 0.04	100	0.07
(Z)-*p*-coumaric acid	29.95	1789.4	1781.9	0.14 ± 0.14	100	0.37*	0.07 ± 0.11	157	–0.01
Protocatechuic acid	30.54	1812.7	1809.5	0.07 ± 0.07	100	0.46*	0.22 ± 0.23	105	–0.01
Quinic acid	31.30	1842.7	1845.6	5.20 ± 7.88	152	0.40*	1.64 ± 0.90	55	–0.37
Gallic acid methyl ester	31.92	1840.0	1875.6	0.63 ± 0.54	86	0.29	0.71 ± 0.61	86	–0.23
(E)-*p*-Coumaric acid	33.00	1947.5	1929.0	0.63 ± 0.56	89	0.48**	0.33 ± 0.30	91	–0.08
Gallic acid	33.29	1945.9	1943.6	15.12 ± 12.20	81	0.54**	23.36 ± 26.6	114	0.08
(E)-Ferulic acid	35.88	2093.1	2079.0	0.15 ± 0.13	87	0.48**	0.20 ± 0.13	65	–0.21
(E)-Caffeic acid	36.68	2135.6	2122.7	0.39 ± 0.40	100	0.52**	1.95 ± 1.77	91	–0.07
4-Hydroxyphenyl-beta-D-glucopyranoside	43.92	2585.4	2562.3	0.39 ± 0.37	93	0.46**	0.18 ± 0.17	94	–0.36
4-Hydroxyphenyl ethyl-b-D-glucopyranoside	46.53		2742.2	2.45 ± 2.64	108	0.40*	0.92 ± 1.26	137	–0.08
(Z)-4-Caffeoyl-quinic acid	47.88	2991.6	2840.2	0.32 ± 0.29	91	0.33	0.58 ± 0.45	78	–0.29
Chlorogenic acid	51.17	3101.6	3093.8	0.03 ± 0.04	133	0.35	0.08 ± 0.26	325	–0.35
Caffeoylquinic acid deriv. 1	51.33		3106.7	0.01 ± 0.01	100	0.44*	0.01 ± 0.01	100	–0.20
Caffeoylquinic acid deriv. 2	51.69		3135.9	0.01 ± 0.01	100	0.52**	0.01 ± 0.01	100	–0.16
(E)-4-Caffeoylquinic acid	51.88	3154.5	3151.5	0.01 ± 0.01	100	0.50**	0.03 ± 0.03	100	–0.31
(E)-5-Caffeoylquinic acid	52.16	3177.6	3174.5	0.01 ± 0.01	100	0.49**	0.04 ± 0.03	75	–0.44
(Z)-1-Caffeoylquinic acid	52.46	3201.0	3199.3	0.04 ± 0.03	75	0.40*	0.06 ± 0.05	83	–0.08
Ellagic acid	53.38	3329.0	3276.8	3.71 ± 3.39	91	0.58**	7.31 ± 7.12	97	0.06
**Lignin and neolignan glycosides**									
(±)-Threo-guaiacylglycerol	34.21		1990.6	0.06 ± 0.05	83	0.01	0.06 ± 0.06	100	–0.02
Guaiacyl deriv. 1	47.43		2807.1	0.05 ± 0.05	100	0.28	0.02 ± 0.02	100	0.07
Guaiacyl deriv. 2	48.83		2911.2	0.35 ± 0.34	97	0.34	0.27 ± 0.33	122	0.07
**Steroids**									
Sterol1	52.24		3181.1	0.18 ± 0.17	94	0.47**	0.07 ± 0.06	86	–0.06
Sterol2	52.91		3237.0	0.14 ± 0.12	86	0.42*	0.16 ± 0.13	81	–0.33
Beta-sitosterol	54.08	3377.2	3337.0	0.04 ± 0.04	100	0.42*	0.03 ± 0.03	100	0.10
**Diterpenes**									
(E)-Phytol	37.32	2169.1	2158.3	0.75 ± 0.76	101	0.47**	0.41 ± 0.25	61	–0.144
**Triterpenes**									
Oleanolic acid deriv. 1	56.40		3544.5	0.04 ± 0.13	325	0.16	0.67 ± 0.66	99	–0.217
Oleanolic acid deriv. 2	57.07		3606.7	0.09 ± 0.10	111	0.19	0.36 ± 0.34	94	–0.284
Oleanolic acid	57.50	3560.0	3647.3	0.58 ± 0.48	83	0.37*	1.08 ± 0.79	73	–0.105
Ursolic acid	58.42	3608.9	3735.6	0.12 ± 0.12	100	0.38*	0.47 ± 0.36	77	–0.021

Concentration values are presented in mg/g DW. RT, retention time; LRI, linear retention index; LRI cal, calculated LRI; SD, standard deviation of mean concentration of compound; r, correlation coefficient; CV, coefficient of variation. *P < 0.05; **P < 0.01.

### Variability Between Species Across All Secondary Metabolites

**FIGURE 2:**
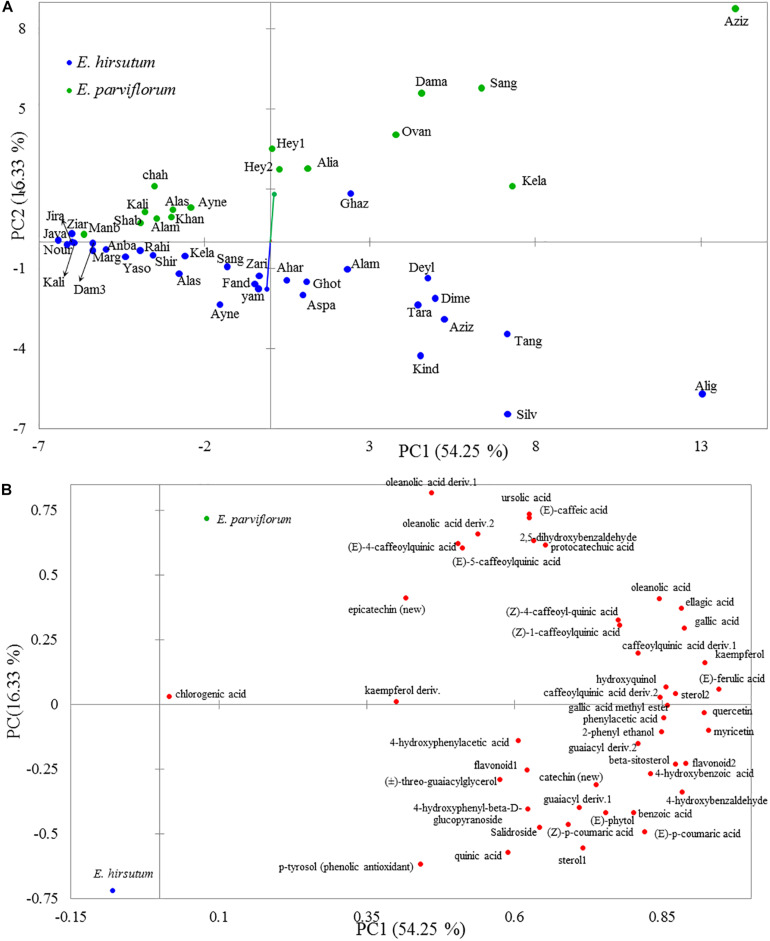
The two-dimensional plot obtained by principal component analysis from 46 secondary metabolites illustrating the variation among *E. hirsutum* and *E. parviflorum* populations **(A)** and the correlation between each chemical variable and the first two principal components **(B)**.

**FIGURE 3:**
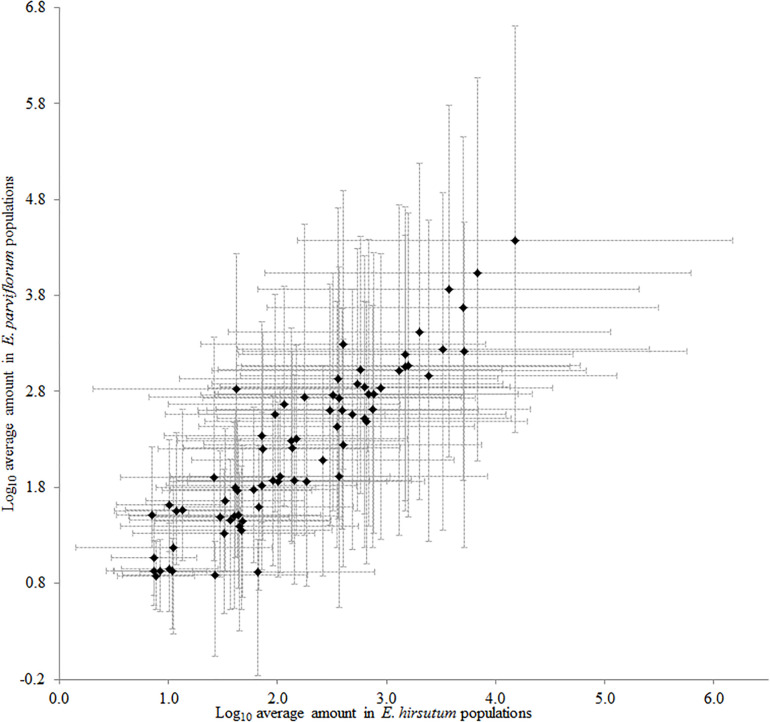
The similarity between *E. hirsutum* and *E. parviflorum* in the amount of 46 secondary metabolites (Pearson’s *r* = 0.91, *R*^2^ = 0.82, *P* < 0.0001).

The two-dimensional plot obtained from principal component analysis revealed a high variation between species, and the second principal component clearly separated most populations of each species (Figure 2). The two main components explained 71% total variation in the chemical compounds. The high contribution of the two first components suggested a considerable correlation between chemical compounds (data not shown). Eight compounds including ursolic acid, oleanolic acid deriv. 1, (E)-caffeic acid, oleanolic acid deriv. 2, (E)-4-caffeoylquinic acid, (E)-5-caffeoylquinic acid, 2,5-dihydroxybenzaldehyde, and protocatechuic acid revealed a positive correlation (loading factor > 0.6), with the second principal component (PC2) represented by populations of *E. parviflorum* (Figure 2). The results of PCA were similar to that including all 74 detected compounds (Supplementary Figure 1). Interestingly, the average level for secondary metabolites was positively correlated between *E. hirsutum* and *E. parviflorum* (*n* = 46, *r* = 0.91, *P* < 0.0001, Figure 3).

### Interspecific Variation in the Level of Flavonoids, Phenolic, Steroids, and Terpenes

**FIGURE 4:**
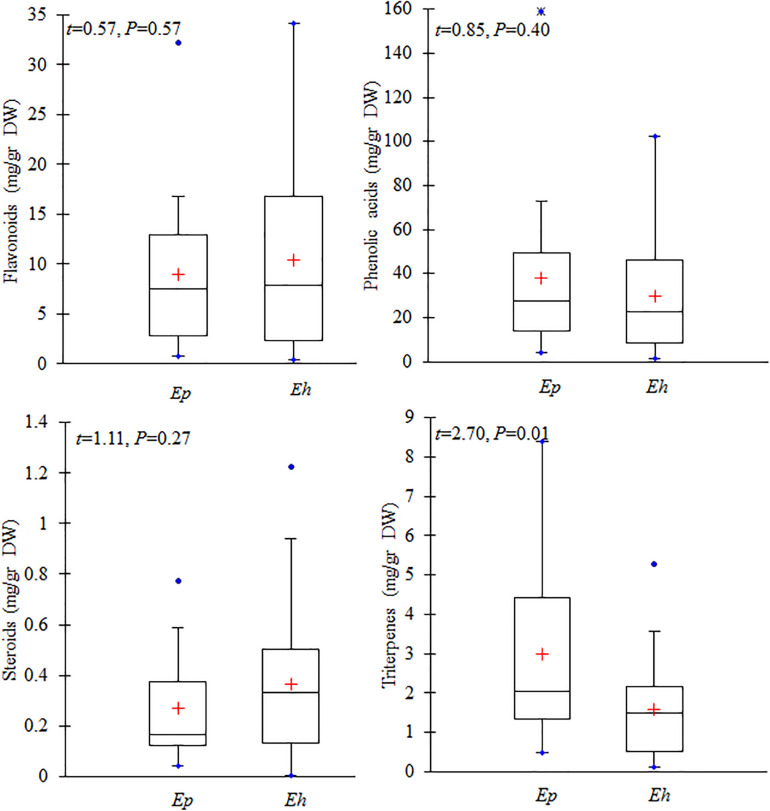
Contents of total flavonoids, phenolic acid and derivatives, steroids, and terpenes in *E. hirsutum* (*Eh*) and *E. parviflorum* (*Ep*) populations. The mean (+), 1st and 3rd quartiles (box), min and max values (blue dot) are shown (*N* = 31 and 16 for *E. hirsutum* and *E. parviflorum*, respectively).

**FIGURE 5:**
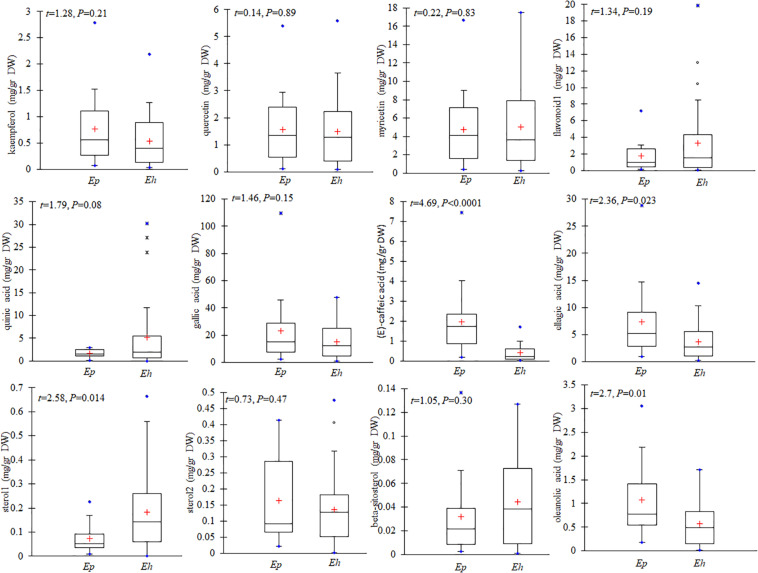
Contents of kaempferol, quercetin, myricetin, flavonoid 1, quinic acid, gallic acid, (E)-caffeic acid, sterol 1, sterol 2, beta sitosterol, and oleanolic acid in *E. hirsutum* (*Eh*) and *E. parviflorum* (*Ep*) populations. The mean (+), median, 1st and 3rd quartiles (box), and min and max values are shown (*n* = 31 and 16 for *E. hirsutum* and *E. parviflorum*, respectively).

All secondary metabolites were classified into four major groups including flavonoids, phenolic, steroids, and terpenes. The mean total amounts of flavonoids (*n* = 8 compounds) in *E. hirsutum* populations (10.46 mg/g DW) was similar to that in *E. parviflorum* (8.90 mg/g DW) (*t* = 0.57, *P* = 0.57, Figure 4). Myricetin was the dominant compound in both *E. hirsutum* and *E. parviflorum* with an average amount of 5.01 and 4.72 mg/g DW (*t* = 0.22, *P* = 0.83), respectively. Catechin was observed in lowest amount in both species (Table 2). On average, the order of the main components of the flavonoids for both *E. hirsutum* and *E. parviflorum* populations was myricetin > flavonoid 1 > quercetin > kaempferol. No significant difference was observed for the main flavonoid compounds between *E. hirsutum* and *E. parviflorum* (Figure 5).

The average total phenolic acid compounds in *E. parviflorum* populations (38.04 mg/g DW) was similar to contents detected in *E. hirsutum* (29.95 mg/g DW, *t* = 0.85, *P* = 0.4, Figure 4). Gallic acid showed the highest concentration among phenolic compounds and also among all other secondary metabolites with a mean value of 15.12 and 23.36 mg/g DW in *E. hirsutum* and *E. parviflorum*, respectively (*t* = 1.46, *P* = 0.15). The other major phenolic components found in *E. hirsutum* were quinic acid and ellagic acid with mean values of 5.20 and 3.71 mg/g DW, respectively (Table 2). Other major phenolic acid components in *E. parviflorum* were ellagic acid, (E)-caffeic acid and quinic acid with mean values of 7.31, 1.95, and 1.64 mg/g DW, respectively. The amount of quinic acid was similar between *E. hirsutum* and *E. parviflorum* populations (*t* = 1.79, *P* = 0.08), conversely ellagic acid (*t* = 2.36, *P* = 0.023) and (E)-caffeic acid (*t* = 4.69, *P* < 0.0001) were significantly greater in *E. parviflorum* than *E. hirsutum* populations (Figure 5).

The total amount of steroid compounds did not vary between *E. hirsutum* (0.36 mg/g DW) and *E. parviflorum* (0.27 mg/g DW, *t* = 1.11, *P* = 0.27, Figure 4). The putatively identified steroids compounds consisted of structurally annotated sterol 1, sterol 2 and β-cytosterol in both *E. hirsutum* and *E. parviflorum*. On average, the amount of sterol 1 was significantly higher in *E. hirsutum* compared with *E. parviflorum* with an average of 0.18 and 0.07 mg/g DW, respectively (*t* = 2.55, *P* = 0.014). β-sitosterol showed the lowest but similar amount in *E. hirsutum* and *E. parviflorum*, with an average of 0.04 and 0.03 mg/g DW, respectively (*t* = 1.05, *P* = 0.30, Table 2 and Figure 4).

The total amount of terpenes in *E. parviflorum* (2.98 mg/g DW) was significantly higher than that detected in *E. hirsutum* (1.58 mg/g DW) (*t* = 2.7, *P* = 0.01, Figure 4). The compounds putatively identified as terpenes consisted of a diterpene, (E)-phytol, and four triterpenes, including structurally annotated oleanolic acid deriv. 1, oleanolic acid deriv. 2, oleanolic acid, and ursolic acid (Table 2 and Supplementary Table 1). Oleanolic acid was the most abundant compound among triterpenes in both *E. hirsutum* and *E. parviflorum* with an average of 0.58 and 1.08 mg/g DW, respectively (*t* = 2.7, *P* = 0.01, Figure 5). The amount of (E)-phytol was higher in *E. hirsutum* (0.75 mg/g DW) as compared to that observed in *E. parviflorum* (0.41 mg/g DW, *t* = 2.30, *t* = 0.027).

### Intraspecific Variation in *E. hirsutum* for Major Groups of Chemical Compounds

A survey of all flavonoids among *E. hirsutum* populations revealed that Alig population contained the highest level of myricetin, quercetin, and kaempferol with 17.5, 5.58, and 2.18 mg/g DW, respectively (Supplementary Table 1). Three populations, Rahi, Ziar, and Java, showed the lowest content (0.03 mg/g DW) of kaempferol and the lowest content of quercetin was also recorded for Java with an amount of 0.09 mg/g DW. The epicatechin showed the highest amount, 0.05 mg/g DW in Alia and Java populations and was found in negligible amount (<0.01 mg/g DW) in 10 *E. hirsutum* populations. Similarly, the highest amount of catechin was recorded for Alig (0.07 mg/g DW) and represented with negligible amount (<0.01 mg/g DW) in 11 (35%) populations. Overall, Alig among the other populations of *E. hirsutum* was rich in flavonoids (Supplementary Table 1).

The results showed that the lowest and highest amounts of total phenolic compounds was found in Java (1.19 mg/g DW, *n* = 26 compounds) and Alig (102.21 mg/g DW), respectively (Supplementary Table 1). The highest amount of gallic acid (47.80 mg/g DW) and ellagic acid (14.53 mg/g DW) was found in Alig population. Two populations Javah and Kali showed the lowest level of gallic acid and ellagic acid (0.91 and 0.22 mg/g DW respectively, Supplementary Table 1). The Silv population exhibited the highest content of quinic acid (30.29 mg/g DW), and interestingly the compound was not detected in Java.

The amounts of steroids and terpenes showed considerable variation among populations. For example, steroids were not found in Java and the highest amount of steroids was observed in Tang and (12.2 mg/g DW, Supplementary Table 1). The content of triterpenes varied from 0.03 mg/g DW (in Kali) to 2.90 mg/g DW (in Gham). The amount of oleanolic acid was remarkable in the Alig population (1.72 mg/g DW) compared to that observed in other populations of *E. hirsutum* (Supplementary Table 1). The amount of (E)-phytol considerably varied from 0.08 mg/g DW in Jira, Ziar, Kale, and Marg to 3.3 mg/g DW in Alig (Supplementary Table 1).

### Intraspecific Variation in *E. parviflorum* for Major Chemical Compounds

Among *E. parviflorum* populations, the highest amount of all flavonoid compounds (32.21 mg/g DW) was observed in Aziz (Supplementary Table 2). Aziz showed the highest content for all four major flavonoid compounds including myricetin, flavonoid 1, quercetin, and kaempferol with 16.65, 7.15, 5.39, and 2.78 mg/g DW, respectively (Supplementary Table 2). The lowest content of main flavonoid compounds (<0.4 mg/g DW) was observed in Manb population. The amount of epicatechin ranged from <0.004 mg/g DW in Chah, Kali, Manb, and Shab to 0.28 mg/g DW in Sang. Similarly, the amount of another additionally detected flavonoid compound, catechin, observed for Alam and Aziz was 0.02 mg/g DW. This compound was found in negligible amounts in several other populations.

Similarly, Aziz population revealed the highest content of the three main phenolic acid compounds including gallic acid, ellagic acid, and (E)-caffeic acid, with 109.55, 28.83, and 7.47 mg/g DW, respectively. The lowest and highest total phenolic compounds among populations of *E. parviflorum*, was found for Manb (4.16 mg/g DW) and Aziz (159.07 mg/g DW), respectively (Supplementary Table 1). The lowest and the highest amount of quinic acid was observed in Kali (0.49 mg/g DW) and Alia (2.99) populations, respectively (Supplementary Table 1).

The lowest and the highest amoun ts of steroids were observed in Manb (0.04 mg/g DW) and Aziz (0.78 mg/g DW) (Supplementary Table 1). Similarly, the lowest and highest amount of triterpenes were observed in Manb (0.38 mg/g DW) and Aziz (7.55 mg/g DW) populations, respectively. In addition, the amount of a diterpene, (E)-phytol, varied from 0.09 (mg/g DW) to 0.84 in Manb and Aziz, respectively (Supplementary Table 1).

### Contribution of Environmental Variables on Phytochemical Variation

**FIGURE 6:**
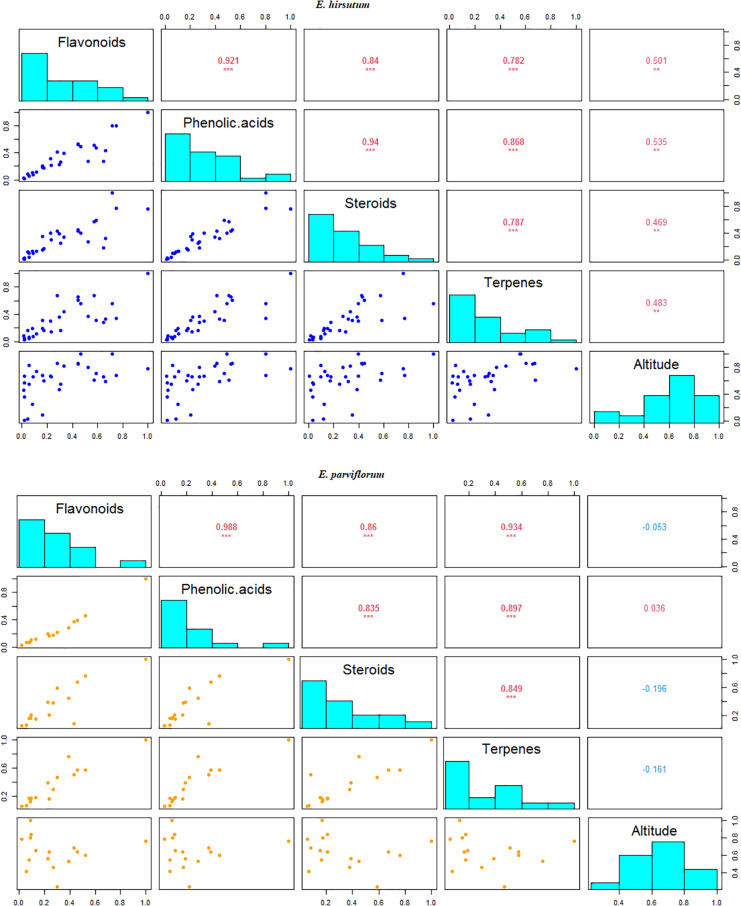
Correlation between the major groups of secondary metabolites and altitude in *E. hirsutum* and *E. parviflorum*. The numbers in upper diagonal represent correlation coefficient; levels of significance at **0.01 and ***0.001.

The results of redundancy combined with variance partitioning analyses exhibited that altitude (adjusted *R*^2^ = 0.17, *P* = 0.001) significantly contributed to the total variation found in the level of secondary metabolites compared to that estimated for climate in *E. hirsutum* (adjusted *R*^2^ = 0.16, *P* = 0.22). In addition, the regression analyses showed that out of 46 secondary metabolites, 34 compounds (74%) showed significant correlation with the altitude in *E. hirsutum* (*P* < 0.05, Table 2). Most of the compounds with a significant correlation, were grouped in flavonoids group (*n* = 6), phenolic acids group (*n* = 21), steroids (*n* = 3), terpenes (*n* = 3). In contrast the redundancy and variance partitioning analyses revealed negligible contribution of both altitude and climatic variable (*P* > 0.05), and none of the compounds revealed a significant correlation with altitude in *E. parviflorum* (*P* > 0.05, Table 2). In addition, the relationship between altitude and four major groups including flavonoids, phenolic acids, steroids, and terpenes were tested separately. Interestingly all pairwise correlations between altitude and these groups were strongly significant in *E. hirsutum* (*r* = 0.48–0.53, *P* < 0.01, Figure 6).

## Discussion

### Natural Variation in the Level of Chemical Compounds

Based on GC/MS analysis and either putative identification or structure annotation, the major compounds in *E. hirsutum* and *E. parviflorum* populations were flavonoids, phenolic acids, steroids, and terpenes. Several studies have also shown that species in *Epilobium* genus are a rich source of secondary metabolites especially polyphenols including flavonoids, phenolic acids, and tannins (e.g., Granica et al., 2014). Some other lipophilic metabolites such as steroids, triterpenoids, and fatty acids have also been detected in various *Epilobium* species (Granica et al., 2014). In our study, myricetin was dominant and constituted the major component of flavonoid group in *E. hirsutum* and *E. parviflorum* populations. Similar studies have reported the occurrence of myricetin as a dominant flavonoid in several *Epilobium* species including *E. hirsutum*, *E. dodonaei*, *E. fleischeri*, *E. roseum*, *E. parviflorum*, *E. montanum*, and *E. tetragonum* whereas quercetin glycosides were a dominant flavonoid in *E. angustifolium* (Slacanin et al., 1991; Ducrey et al., 1995; Hiermann, 1995; Hevesi Tóth et al., 2006; Granica et al., 2014). In this study, the order of the most abundant flavonoids was similar (myricetin > flavonoid 1 > quercetin > kaempferol) in both *E. hirsutum* and *E. parviflorum*. In a similar study, myricetin, quercetin, kaempferol, and their various glycosides were dominant in *E. parviflorum* (Hevesi Tóth et al., 2009).

The similar level of total flavonoids in *E. hirsutum* and *E. parviflorum* in our study is consistent with the results obtained by a photometric method exhibiting no significant difference between five *Epilobium* species (varies from 7.3 to 8.3 mg/g herb among *E. parviflorum* Schreb., *E. roseum* Schreb., *E. tetragonum* L., *E. montanum* L., *E. angustifolium* L.) from Hungary (Hevesi Tóth, 2009). Although the average flavonoid content observed for our study species was slightly higher than that reported in Hevesi Tóth (2009), some populations of *E. hirsutum* (Alig, 34 mg/g DW) and *E. parviflorum* (Aziz 32 mg/g DW) showed three times higher concentrations compared with those observed in species from Hungary.

Gallic acid showed the highest amount among all phenolic compounds with 35 and 46% of all secondary metabolites detected in both *E. hirsutum* and *E. parviflorum*, respectively. Although there was no significant difference in the total level of phenolic compounds, quinic acid, and gallic acid between species, the level of ellagic acid and (E)-caffeic acid was significantly different between species. Contrary to our study, the level of polyphenols was two times higher in *E. hirsutum* compared to that reported in *E. parviflorum* in a population from Estonia (Remmel et al., 2012). However, similar to our results, the level of gallic acid was similar between species and the amount of ellagic acid was significantly higher in *E. parviflorum* than that in *E. hirsutum* (Remmel et al., 2012). Ellagic acid is a marker compound indicating the presence of ellagitannins consistent with earlier reports in five species of *Epilobium* including the most popular *E. angustifolium*, *E. hirsutum*, and *E. parviflorum* (Slacanin et al., 1991; Barakat et al., 1997; Nawwar et al., 1997; Kiss et al., 2004; Hevesi Tóth et al., 2006; Shikov et al., 2006; Stolarczyk et al., 2013; Granica et al., 2014; Monschein et al., 2015). Thus, our results suggest that *E. parviflorum* populations might also contain higher contents of ellagitannins compared to *E. hirsutum* populations due to high amounts of ellagic acid. The antioxidant capacity of plant extracts of *Epilobium* has generally been associated with high levels of phenolic compounds (Katalinic et al., 2006; Wojdyło et al., 2007; Hevesi Tóth et al., 2009). Thus, our results indicate that Alig (*E. hirsutum*) and Aziz (*E. parviflorum*) populations might also exhibit the highest antioxidant capacity because of rich phenolic compounds compared with other populations.

### Additionally Detected Compounds and Medicinal Prospects

In this study, the large-scale geographic investigations on the natural populations from both species provide opportunity for the putative identification of additionally detected chemical compounds not previously reported including catechin, epicatechin, terpenes, allantoin, and steroids. Two flavonoid compounds including catechin and epicatechin were detected in low level in most populations of *E. hirsutum* and *E. parviflorum* and are reported in this study for the first time. All other putatively identified flavonoid compounds except catechin and epicatechin have also been reported in most species of *Epilobium* (Granica et al., 2014). Although the total mean values of terpenes in *E. parviflorum* was significantly higher than *E. hirsutum*, triterpenoid acids have only been detected in the aerial part of *E. angustifolium* and are not reported from other *Epilobium* species (e.g., Glen et al., 1967; Granica et al., 2014). Therefore, the result of our study is interesting because of the detection and semiquantification of terpenes (diterpenes and triterpenes) in Iranian populations of *E. hirsutum* and *E. parviflorum*.

Several steroid compounds including cholesterol, campesterol, stigmasterol, β-sitosterol, and its glycosides and esters have been reported in *E. angustifolium*, *E. parviflorum*, and *E. obscurum*, which are not found in other species of *Epilobium* (Haddock et al., 1982; Hiermann and Mayr, 1985; Nowak and Krzaczek, 1998; Granica et al., 2014). In our study, the presence of β-sitosterol in *E. hirsutum* populations is reported for the first time. Considering the high yield of shoot biomass, a higher level of metabolites is expected in *E. hirsutum* compared with *E. parviflorum* (Constantin et al., 2013).

Allantoin has not been reported in any studies on *Epilobium* species, and it is detected in most populations of *E. hirsutum* and *E. parviflorum*, in our study. Allantoin has several beneficial effects as an active ingredient in cosmetics including its protective effect on the skin and moisturizing and keratolytic effects, promoting cell proliferation and wound healing, as well as anti-irritant and skin-protecting effects through the formation of complexes with sensitizing and stimulating agents (Araújo et al., 2010). According to the reports, a soft lotion with 5% allantoin improves recovery of inflammation and the wound-healing process, and quantitative analysis supports the idea that allantoin promotes proliferation of fibroblasts and synthesis of extracellular matrix (Araújo et al., 2010). Similarly, treatment of itching in mild-to-moderate atopic dermatitis is also reported with a topical steroid drug containing allantoin (Veraldi et al., 2009). In addition, allantoin is often used in toothpaste, mouthwash, and other oral hygiene products in shampoos, lipsticks, anti-acne products, sun care products, clarifying and cleansing lotions, various makeup lotions and creams, and other cosmetic and pharmaceutical products (Thornfeldt, 2005). One of the reasons that *Epilobium is* effective in the treatment of skin and mucosa diseases as well as bodily injuries might be due to the presence of allantoin. On the other hand, the leaf and flower extract of the plant is effective for many skin problems such as eczema, acne, skin rashes, minor burns, and ulcers. North American Indians have used *Epilobium* species to treat infected wounds, hemorrhages after parturition, and swelling (Smith, 1923; Barrett and Gifford, 1933; Bocek, 1984; Granica et al., 2014). Thus, due to its high shoot biomass and easier seed and clonal propagation especially for *E. hirsutum*, this plant can be used as a putative natural source to extract several compounds including catechin, epicatechin, and allantoin.

### Inter- and Intraspecific Variability and Environmental Clines

Although almost all compounds were found in both species in reasonable amounts, our study revealed extensive natural variation among species and within each species (high variation in CV for various compounds). Our results showed that the level of chemical compounds are correlated between species, suggesting similarity in metabolic pathways between species (Wink, 2003). In addition, the results also show that several compounds within each species are tightly correlated together, indicating a common genetic basis including genes with pleiotropic effect affecting multiple traits simultaneously.

Although climatic factors have been major drivers of genetic differentiation among populations for various species (Castilla et al., 2020; Gibson and Moyle, 2020), bioclimatic variables exhibit less profound effect on the level of chemical variation in our study. However, altitude is found to be a major environmental factor affecting the level of secondary metabolites by explaining 17% of total variation found in *E. hirsutum*. Accordingly, the results showed a significant relationship between the amount of chemicals in two-thirds of secondary metabolites and altitude in *E. hirsutum*. One reason to explain the negligible effect of altitude on secondary metabolites in *E. parviflorum* compared with *E. hirsutum* may be due to a narrower altitudinal range in 641–2,670 m a.s.l. in the former relative to a wider geographic range surveyed for the latter species (14.6–2600 m a.s.l.). In a similar study, the concentration of quercetin and flavanol was correlated with altitude in *E. angustifolium* (Monschein et al., 2015). It has also been suggested that plants respond to cooler temperature and other environmental stimuli including UV radiation by increasing production of flavonoids (Jaakola and Hohtola, 2010). Congruent to our results obtained from *E. hirsutum*, the correlation between altitude and phenolic and terpenoid compounds has also been reported in other studies (e.g., Martz et al. (2009). Hence, the observed altitudinal effects on the level of flavonoids, phenolic acid, steroids, terpenes, and allantoin suggest that the production of these compounds as potential markers can significantly be manipulated in *E. hirsutum* populations. In addition, these results suggest that other environmental variables correlated with altitude including temperature and UV-B are likely to influence the amount of chemical compounds. Moreover, to determine the specific factors affecting the chemical compounds, further laboratory investigations are required.

## Conclusion

This research is the first study that applies a comparative approach by including multiple populations from two congeneric species to evaluate intra- and interpopulation phytochemical variation for *E. hirsutum* and *E. parviflorum*. The wide geographic sampling revealed additionally detected compounds such as catechin, epicatechin, terpenes, and allantoin in both species and steroids in *E. hirsutum* which have not been reported previously. While the level of secondary metabolites was correlated between species suggesting similarity in metabolic pathways in congeneric species, each compound exhibited a considerable variation among populations with species. In addition, we found that the populations of two species were differentiated in few compounds including oleanolic acid deriv. 1, ursolic acid, and (E)-caffeic acid as demonstrated based on correlation with the second principal component of PCA splitting most congeneric populations from each other. The effect of climate based on 19 bioclimatic variables to explain the total variation was not significant in both species. However, altitude exhibited a significant contribution on total chemical variation, and the four major groups of secondary metabolites were also significantly correlated with altitude in *E. hirsutum*. We also found significant positive correlation between altitude and most of the individual compounds from flavonoids, phenolic acids, steroids, and terpenes mainly in *E. hirsutum*. These results suggest that other biotic or abiotic factors that are dependent on altitude might affect the production of specialized metabolites, thus further experiments can elaborate these factors in more details.

## Data Availability Statement

Metabolomics raw data have been deposited to the GNPS database (Global Natural Products Social Molecular Networking, Wang et al. (2016)) with the identifier: MassIVE MSV000086585. The complete dataset can be accessed here - doi: 10.25345/C5Z19Z.

## Author Contributions

MMB designed and planned the study. MMB, MF-A, and JR conducted the field sampling, laboratory, and data analyses. MMB wrote the manuscript and all co-authors contributed to the revision. All authors contributed to the article and approved the submitted version.

## Conflict of Interest

The authors declare that the research was conducted in the absence of any commercial or financial relationships that could be construed as a potential conflict of interest.

## Supplementary Material

Supplementary Figure 1 The two-dimensional plot obtained from all 74 compounds detected in the extracts of *Epilobium hirsutum* and *E. parviflorum*. The first two principal components explained 67% of all variation found in the chemical compounds.

Supplementary Table 1 Chemical composition (mg/g DW) of 31 and 16 natural populations of *Epilobium hirsutum* and *E. parviflorum*, respectively.

Supplementary Table 2 Bioclimatic variables extracted from WorldClim 2 databases for natural populations of *Epilobium hirsutum* and *E. parviflorum*.
