# Supplementary figures and images for: Comparative Analyses of Phytochemical Variation Within and Between Congeneric Species of Willow Herb, Epilobium hirsutum and E. parviflorum: Contribution of Environmental Factors
Source: Front Plant Sci. 2021 Feb 17;11:595190. doi: 10.3389/fpls.2020.595190 (PMC7925418; doi:10.3389/fpls.2020.595190)

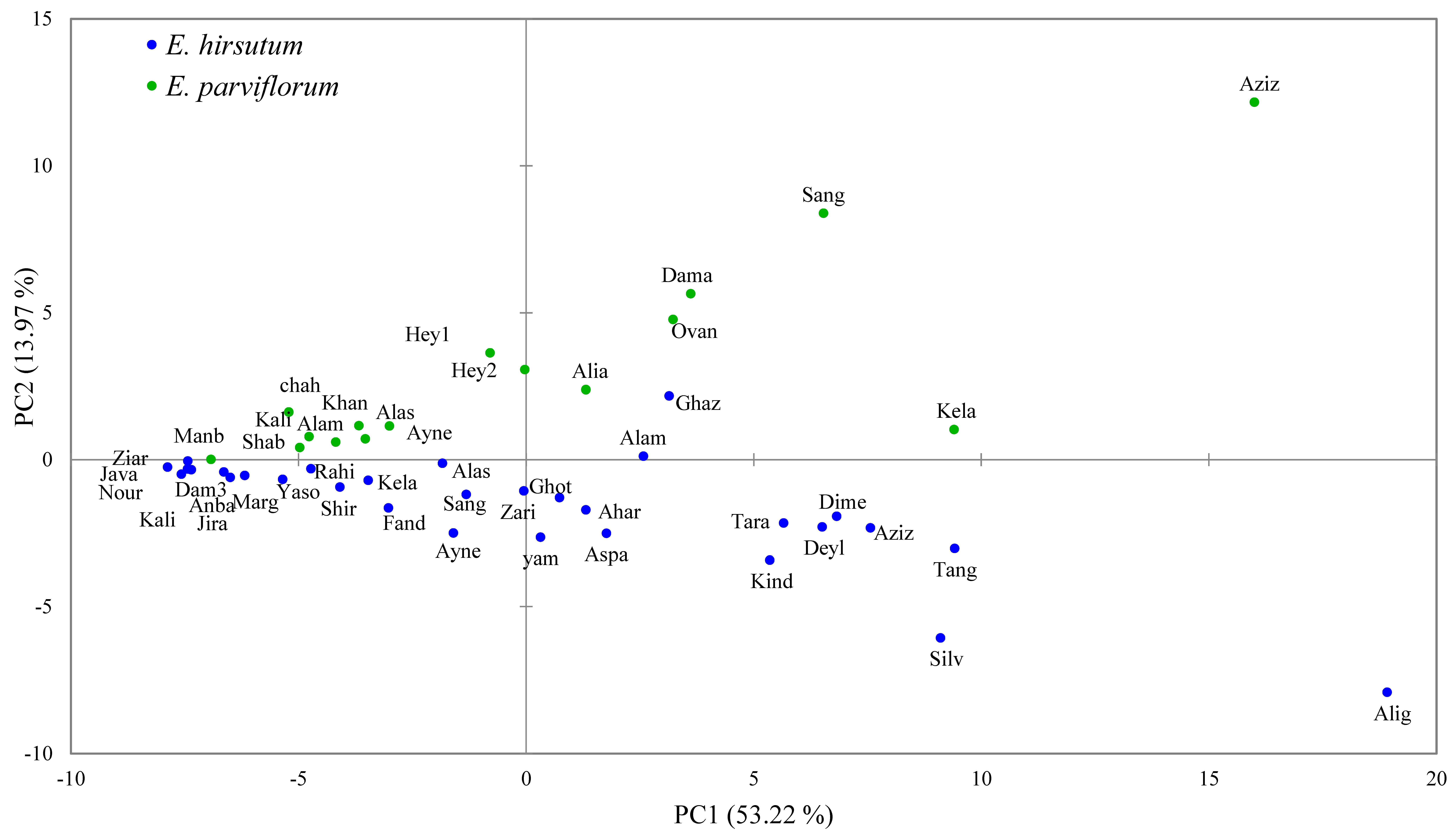

Supplement: Supplementary Figure 1 — The two-dimensional plot obtained from all 74 compounds detected in the extracts of Epilobium hirsutum and E. parviflorum. The first two principal components explained 67% of all variation found in the chemical compounds. [file Image_1.tif]
